# *Ilex paraguariensis* Extract Increases Lifespan and Protects Against the Toxic Effects Caused by Paraquat in *Caenorhabditis elegans*

**DOI:** 10.3390/ijerph111010091

**Published:** 2014-09-26

**Authors:** Maria E. Lima, Ana C. Colpo, Willian G. Salgueiro, Guilherme E. Sardinha, Daiana S. Ávila, Vanderlei Folmer

**Affiliations:** 1 Programa de Pós Graduação em Bioquímica, Universidade Federal do Pampa, BR 472-Km 592-Caixa Postal 118-CEP: 97500-970-Uruguaiana-RS, Brazil; E-Mails: mduda_89@hotmail.com (M.E.L.); anaccolpo@hotmail.com (A.C.C.); avilads1@gmail.com (D.S.A.); 2 Faculdade de Farmácia, Universidade Federal do Pampa, BR 472-Km 592-Caixa Postal 118-CEP: 97500-970-Uruguaiana-RS, Brazil; E-Mails: willian_cgs@hotmail.com (W.G.S.); guisardinha@gmail.com (G.E.S.)

**Keywords:** paraquat, oxidative stress, *Ilex paraguariensis*

## Abstract

Recent studies have shown that phenolic compounds present in yerba mate have antioxidant defense properties. To verify whether *Ilex paraguariensis* extracts are capable of increasing the lifespan of an organism, we have used the free-living nematode *Caenorhabditis elegans*. Notably, this is the first study that analyzes the effects of the extracts of yerba mate obtained from an extraction method that mimics the manner that the plant is consumed by the population by using a live organism. Yerba mate was purchased from commercial markets from Argentina, Brazil, and Uruguay. *Ilex paraguariensis* extracts significantly increased the life span of *C. elegans*. Moreover, the extracts reduced the ROS levels *per se*, and protected from the reduced survival and reproduction rate induced by paraquat exposure. Considering molecular aspects, we observed that the worms pretreated with the extracts depicted higher translocation of the transcription factor DAF-16::GFP to the nucleus. However, there was no increase in the levels of the DAF-16 target genes, SOD-3 and catalase. Our results suggest that the increase of lifespan caused by the different extracts is associated to the antioxidant potential of yerba mate, however this effect is not completely mediated by *daf-16*.

## 1. Introduction

*Ilex paraguariensis*, popularly known as yerba mate, is a tree species native from South America which is used to prepare a peculiar drink. In countries as Argentina, Brazil, Uruguay and Paraguay, where it is traditionally consumed, it receives different names such as “mate”, “tererê” or “chimarrão” [1,2]. Among the bioactive compounds present in yerba mate, there are phenolic compounds, saponins, xanthines, minerals and vitamins. The antioxidant, antiinflammatory, antimutagenic, anti-glycation and weight reduction properties of these compounds are well documented [3,4,5].

Recently, there has been great interest in studying natural compounds that assist in preventing and/or maintaining a healthy lifespan (“healthspan”) in humans. However, humans and mammals in general have long lifespans and hence, longevity studies are difficult to assess in these models. In this context, *C. elegans* emerges as an alternative model that enables well-characterized toxicological and pharmacological studies. *C. elegans* is a free-living nematode, found in soil rich in organic matter. Experimentally, it has the advantage of being a simple animal, with a nervous, reproductive and digestive systems present in a small worm (adults are ∼1.2 mm in length). Other advantages include rapid life cycle and short lifespan, which are relevant for lifespan investigations; and the possibility of using fluorescent markers with genes of interest, which allows protein expression to be observed in live animals [6,7]. In addition, *C. elegans* is also used as model system to investigate the molecular pathways involved in the pathogenesis of neurodegenerative diseases such as Parkinson’s and Alzheimer [8,9]. This fact is important because these diseases are specific to mammals, and in this respect the predictability of the results observed in worms to mammals is very relevant in toxicology and pharmacology. This predictability is due to the high genetic homology between humans and *C. elegans* (about 70%) [10].

Among these genes is *daf*-16, which encodes a homolog of the human FOXO transcription factor. DAF-16 acts towards insulin/IGF-1 signaling, responsible for regulating various biological processes such as longevity, lipid storage, reproduction, stress response, thermotolerance, resistance against pathogens, metabolism and autophagy [11]. This gene is regulated by a surface receptor, DAF-2 [9]. When an insulin-like peptide binds to DAF-2, it triggers a cascade of phosphorylation that starts by AKT phosphorylation and this in turn phosphorylates DAF-16, thereby preventing its translocation to the nucleus. When this response is prevented, by removal of the ligand or by inhibition, DAF-16 remains dephosphorylated and translocates to the nucleus where it activates the transcription of longevity genes, as *sod-3 ctl-1* and *2*, which in turn will encode the transcription of antioxidant enzymes such as superoxide dismutase (SOD) and catalase (CAT) [10,11].

In a previous study we investigated yerba mate marketed in Brazil [12], Argentina and Uruguay and we analyzed how sequential extractions, that occur when the infusion is taken, may influence the concentration of the compounds extracted and the *in vitro* antioxidant capacity of the beverage. We conclude that the compound concentrations can vary, however the antioxidant capacity is maintained at significant levels. To improve our knowledge about how these variations can mediate cellular responses we designed this study. Our hypothesis is that the total polyphenol content present in yerba-mate extracts can affect the lifespan and protect against the toxic effects caused by paraquat in *Caenorhabditis elegans.* Based in the considerations cited above, this study aimed:

(I) To verify whether there is some degree of toxicity of the extracts of *Ilex paraguariensis*, particularly their effectson the lifespan of the nematode *C. elegans*;
(II) To analyze whether the extracts of *Ilex paraguariensis* can cause changes in reproduction and/or locomotor activity of *C. elegans*;
(III) To investigate whether the extracts of *Ilex paraguariensis* protect from the toxic effects induced by paraquat exposure;
(IV) To investigate whether the insulin/IGF-1 pathway is involved in these effects.

## 2. Experimental Section

### 2.1. Chemicals

All reagents were obtained from Sigma (St. Louis, MO, USA) or from local suppliers.

### 2.2. Yerba-Mate

To develop this study we used herbs purchased in local markets. Three different brands acquired in Argentine, three in Brazil and three in Uruguay. The chosen herbs are considered “traditional” presentation forms. The herbs were named Ar for Argentine brands; Br for Brazilian brands and Uy for Uruguayan brands.

### 2.3. Extraction Method

**Figure 1:**
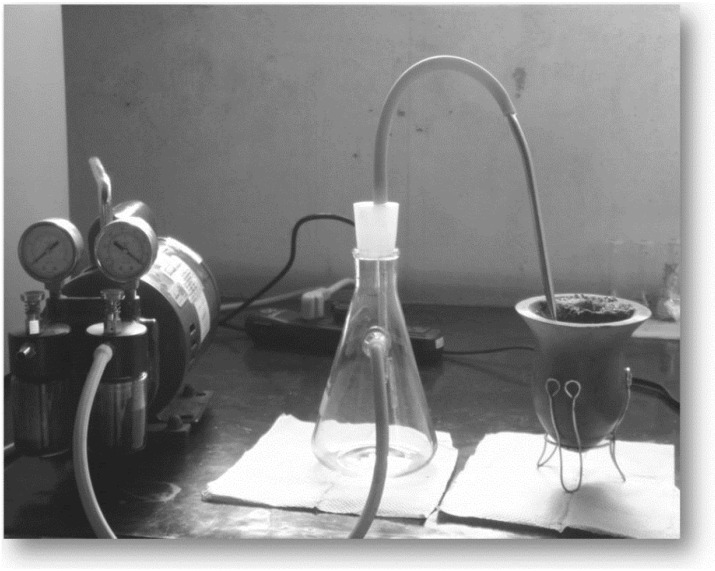
Extraction method—mimicking the “mate” preparation.

The aqueous extracts were obtained by an extraction process that mimics the “chimarrão” preparation (as can be seen in Figure 1), by addition in a medium size gourd, of a sufficient amount of yerba to occupy one third of the volume of the bowl (85 g), and the free volume was completed with water (70 mL) at 80 °C. The water in the bowl remained in contact with the herb for 1 minute and right after the water was sucked through a “pump” attached to a suction system. The extracts from the 1st, 2nd, 5th, 10th and 15th infusions (mate) were filtered, and stored for subsequent experiments. The 15th infusion is equivalent to the 1L amount of water usually consumed by people taking mate.

### 2.4. Strains and Growth Conditions

*C. elegans* strains were routinely propagated at 22 °C on Nematode Growth Medium (NGM) plates containing a lawn of *Escherichia coli* strain *OP50* as a food source [13]. Strains used in this study were: N2 (var. Bristol); TJ356, zls356[(Pdaf-16::daf-16::GFP);GA800,wuIs151[(ctl-1+ctl-2+ctl-3;myo-2::GFP]) and CF1553,muIs84([Psod-3::GFP]). The strains and the bacterium *Escherichia coli* OP50 were obtained from the *Caenorhabditis* Genetics Center (Minessota, EUA).

### 2.5. Extract Pretreatment

Synchronized L1 worms (2000) were treated at 22 °C for 30 min by constant agitation in a rotator with each of the extracts of *Ilex paraguariensis*, of different origin, followed by three washes with 85 mM NaCl solution at the end of the incubation. Worms were placed on OP50-seeded NGM plates and the number of surviving worms on each plate was counted 24 h after exposure.

### 2.6. Paraquat Exposure

To induce toxic effects in *C. elegans*, 2000 synchronized L1 worms were exposed for 30 min to paraquat (Gramoxone 200^®^), a pro-oxidant agent, at the concentration of 1 mM, right after the pre-treatment with *Ilex paraguariensis* extracts, followed by three washes with 85 mM NaCl solution at the end of the incubations. Afterwards, worms were transferred to NGM plates and 24 h after treatments the live worms were counted. The experiment was repeated three times. All those worms seen moving on the agar were considered alive. We plotted a curve with different doses of paraquat (0.1, 1, 3, 5, 7 mM). The dose of 1 mM killed 50% of the worms (data not shown). For this reason, we have selected this concentration for our analysis.

### 2.7. Egg Laying

Following 24 h of acute exposure to extracts of *Ilex paraguariensis* only or to extracts followed by paraquat, worms were individually transferred to new NGM plates seeded with *OP50*. For assessing egg laying, nematodes were monitored and transferred to a new plate every 1.5 days, and the total number of eggs released on the plates was scored [13]. The data were expressed as percent of control. The experiments were repeated in triplicate in three independent worm preparations.

### 2.8. Behavior

The behavioral parameter was performed to evaluate the motor activity of the worms treated only with extracts of *Ilex paraguariensis*. For motor activity, the L1 recent treated animals were transferred to a new NGM plate without *OP50*, acclimatized for 1 min and then the number of times that the animal moved its head upwards during the movement was counted for 1 minute (head trashes).

### 2.9. Lifespan Experiments

We chose to test the 1st and 15 th best extracts of *Ilex paraguariensis* from each origin in the N2 strain (Bristol). After the acute exposure to *Ilex paraguariensis* extracts, live and healthy-looking worms (around 20 per condition; in duplicates) were collected at the same day at the late L4 stage and transferred every 2 days to new *OP50*-seeded NGM/5-fluoro-2’-deoxyuridine (FUDR, Sigma) plates. Survival was assessed daily until all the worms were dead. Each experiment was repeated in triplicate in three independent experiments.

### 2.10. ROS Measurements 

Worms exposed to the extracts of *I. paraguariensis* were washed two additional times in saline buffer and were transferred to a 96-well plate and 2’7’ dichlorofluorescein diacetate (DCF-DA) was added at a final concentration of 3.25 mM and the fluorescence levels were measured (excitation: 485 nm; emission: 535 nm), as previously described by Liao *et al*. with modifications, using a CHAMELEON™V Hidex Model 425-106 plate reader [14]. The fluorescence from each well was measured for 120 min at 10 min intervals. Fluorescence measurements were normalized to time zero values and rates of increase or decrease in fluorescence (reflecting ROS levels) were expressed as percent control. The experiments were performed in triplicate for three independent worm preparations.

### 2.11. Fluorescence Quantification

After the pre-treatment with different extracts the GFP-expressing strains (CF1553 [muls84], GA800 [wuls154]) were transferred right after the end of the washes into 200 µL saline buffer in a well of 96-well plate. Total GFP fluorescence was measured using 485 nm excitation and 530 nm emission filters. Overall GFP fluorescence of GFP-expressing populations was assayed using a CHAMELEON™V Hidex Model 425-106 plate reader. All experiments were done in duplicate and repeated at least four times.

### 2.12. Statistical Analysis

For survival and longevity analysis, we performed a dose-response curve and plotted a sigmoidal dose response curve using nonlinear regression followed by a Bonferroni *post hoc* test. For the other analysis one-way ANOVA followed by a *post hoc* Dunnet test was performed. For all the experiments, the effects of *I.paraguariensis* treatment were compared to untreated controls assayed in parallel. *P* values <0.05 are considered statistically significant. In all figures, error bars represent the standard error of the mean.

## 3. Results and Discussion

The present study showed for the first time that extracts of yerba mate from different origins protected the nematode *C. elegans* from the toxic effects caused by paraquat and extended worms’ lifespans *per se*. These beneficial effects of the extracts might be correlated with their high levels of polyphenols and methylxanthines, as shown by a previous study from our group and presented in Table 1 [12].

**Table 1:** Total phenolic compounds in sequential extractions performed with herbs of different origins, expressed in mg of gallic acid equivalents per mL of aqueous extract. This previous study analyzed the extracts of *Ilex paraguariensis* used in this manuscript [12].

Herbs	I1	I2	I5	I10	I15
Ar	0.711 ± 0.15	0.967 ± 0.32	0.694 ± 0.16	0.359 ± 0.04	0.221 ± 0.08
Br	0.798 ± 0.19	0.599 ± 0.14	0.459 ± 0.06	0.338 ± 0.21	0.191 ± 0.05
Uy	1.033 ± 0.05	1.451 ± 0.21	1.233 ± 0.13	0.547 ± 0.24	0.514 ± 0.14

Many effects of *Ilex paraguariensis* have been already well described in the literature, especially the antioxidant capacity of the extracts [1]. Most of these studies with yerba mate are performed by isolating some of its main constituents or by preparation of ethanolic extracts, which do not represent the way the plant is consumed. Thus, the present study evaluated commercial yerba mate samples and sought to perform the extractions in a similar manner as the product is prepared and consumed by the population. In southern Brazil, Argentina and Uruguay, “chimarrão” consumption is seen as a social habit where people engage in a round table conversation while enjoying its bitter taste. Considering the culture and the relevance of its use, studies such the present one that evaluates *in vivo* the effects of *Ilex paraguariensis*, may help to transpose to the population the knowledge of its biochemical and pharmacological relevance.

### 3.1. Ilex paraguariensis Extracts Did Not Cause Toxic Effects in C. elegans

Many researches have been conducted with *C. elegans* to examine whether fruit and plants used by the population may exert beneficial effects in this model [14,15,16]. A classic example was reported by Zarse *et al*. [17], who used a compound isolated from green tea, L-theanine, and found that treatment with this compound increased significantly the lifespan of *C. elegans*. Similarly, Di Fan *et al.* analyzed the effects of the extract of spinach in the lifespan, thermal and oxidative stress in *C. elegans*, and verified that the extract increased the survival against the two types of stress [18]. The consumption of antioxidants from vegetables that may be acquired by the diet is thus ideal.

**Figure 2:**
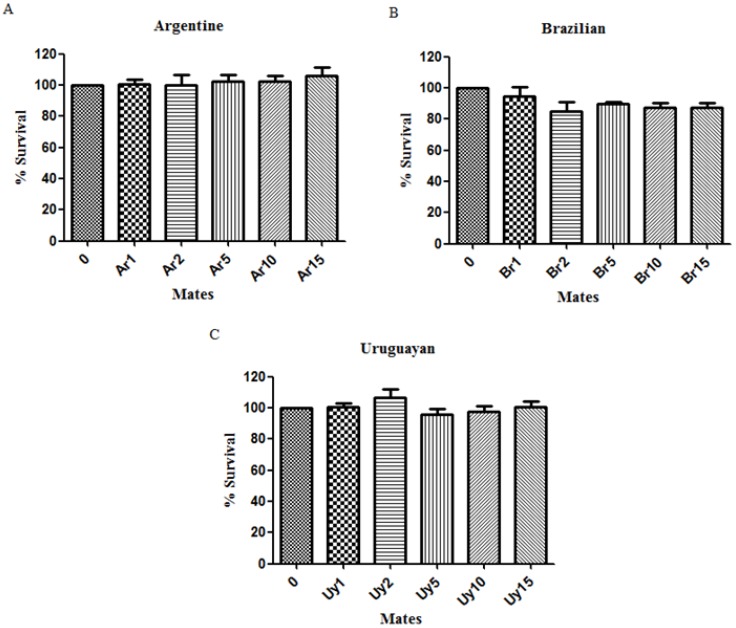
Percentage of surviving worms after acute exposure to extracts of *Ilex paraguariensis* or control (NaCl). (**A**) Argentine brands; (**B**) Brazilian brands; (**C**) Uruguayan brands. There was no difference between the survival of treated compared to control worms in any of the tested herbs. Data are expressed as mean ± SEM.

Initially, we verified whether our extracts were toxic to this nematode and as we demonstrate in Figure 2, the acute exposure to the different extracts did not alter the survival rate of *C. elegans*. In a pilot test we evaluated the five extracts that we prepared and stored, however as we did not observe toxic effects, so we decided to study the first (and the most concentrated) and the fifteenth (the most diluted) extracts of *Ilex paraguariensis*. In addition, these extracts did not change the motor activity of the worms, as the number of head trashes was not altered (data not shown).

### 3.2. Ilex paraguariensis Extracts Exert a Protective Activity against the Toxic Effects Induced by Paraquat in C. elegans

**Figure 3:**
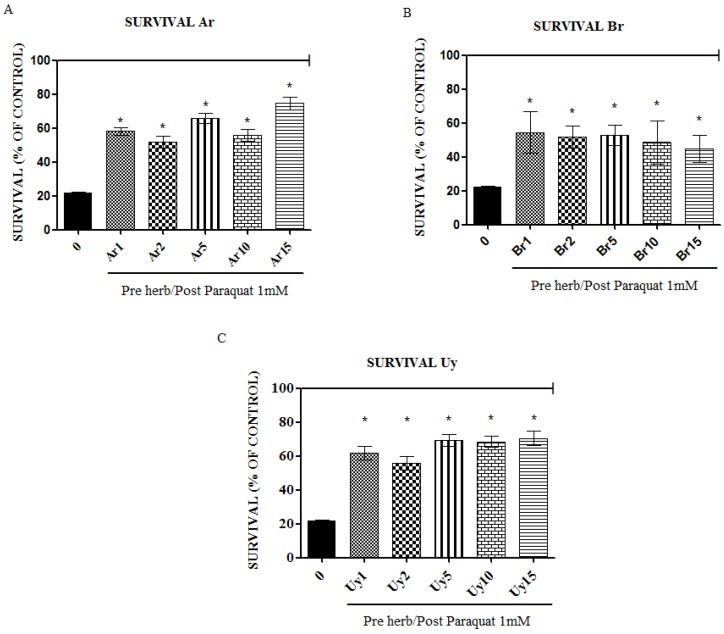
*Ilex paraguarensis* extracts increase the survival of *C. elegans* in contact with paraquat-induced oxidative stress. The line indicates the control group (100%). (**A**) Argentine brands; (**B**) Brazilian brands; (**C**) Uruguayan brands. Data are expressed as mean ± SEM. * indicates statistical difference (*p* < 0.05) from PQ (1 mM) group.

To examine whether the extracts of *I. paraguariensis* could exert protective effect against induced oxidative damage in *C. elegans,* we used paraquat, a known superoxide generator to cause toxic effects in *C. elegans* [19]. Our results showed that all extracts were able to protect the nematode *C. elegans* from the mortality induced by paraquat. The survival rate of worms pre-treated with extracts was of approximately 20 to 60% higher when compared to untreated ones, as can be seen in Figure 3.

**Figure 4:**
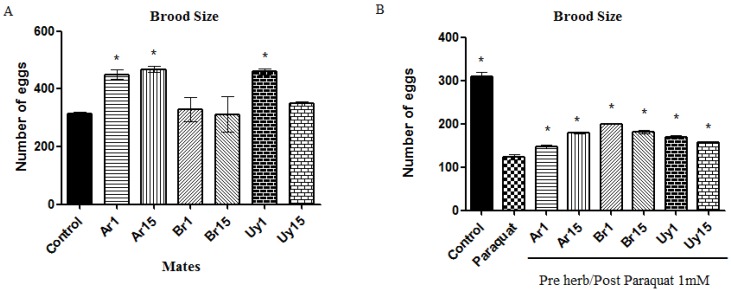
Reproduction of worms pre-treated with *Ilex paraguariensis* and exposed to paraquat (1 mM). (**A**) Worms exposed to only herbs; (**B**) Worms pre-exposed to herbs/post-exposed to paraquat. Data are expressed as mean ± SEM. * indicates *p* < 0.05 as compared to control (A) or paraquat (B).

In addition, the extracts prevented the egg laying reduction induced by paraquat in the worms, by increasing the mean of the total egg laying in comparison to the paraquat group (Figure 4A,B). We believe that these results can be partially explained by the amount of phenolic compounds present in the extracts that should be exerting an antioxidant activity *per se* [14].

### 3.3. Ilex paraguariensis Extends C. elegans Lifespan

According to the free radical theory of aging, oxidative stress is directly related to the reduction of the lifetime in model organisms [18]. Considering that oxidative stress accelerates the aging processes and the antioxidant properties of *I. paraguariensis* that has been described in the literature, we evaluated the effects of extracts (1st and 15th) on *C. elegans* lifespan.

**Figure 5:**
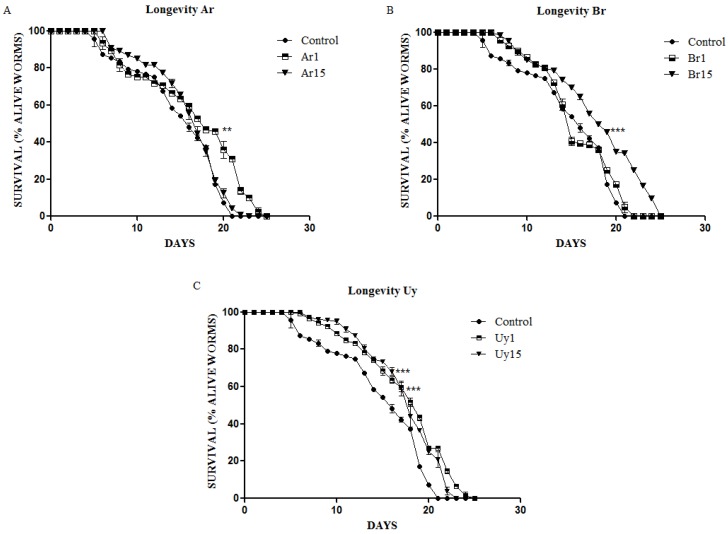
Lifespan of worms treated with extracts (I1 and I15). (**A**) Argentine herb; (**B**) Brazilian herb; (**C**) Uruguayan herb. Data are expressed as mean ± SEM. ** indicates *p* < 0.05 and *** *p* < 0.001 as compared to control group.

This study showed for the first time that treatment with extracts of *I. paraguariensis* was able to extend the average lifespan of the nematode *C. elegans*. Some extracts with lower content of polyphenols also significantly increased the lifespan in *C. elegans* [11]. Our results show that the extracts Uy1, Uy15, Ar1and Br15 (Figure 5) were able to increase significantly the lifespan of *C. elegans* when compared to control.

Saul *et al.* analyzed the effects of four polyphenols derived from plants on *C. elegans* lifespan, stress resistance, behavior, among other endpoints, and found that all four compounds—tannic acid (TA), gallic acid (GA), ellagic acid (EA), and catechin (CT)—increased the lifespan of this nematode [19]. Similarly, our extracts, which have considerable amounts of polyphenols, also increased the average lifespan of *C. elegans*. According to these results, we believe that the phenolic composition of our extracts is related to the extension of the mean lifespan in *C. elegans*.

In contrast, some studies showed some association between oxidative stress and the lifespan of *C. elegans* [20,21]. Ristow *et al.* concluded that small stress stimuli, such as caloric restriction and glucose restriction, enhanced mitochondrial activity and subsequently increased ROS formation [20]. The authors considered that this induces an adaptive response and increased stress defenses, improving metabolism and increasing the longevity of *C. elegans*. Furthermore, Pun *et al.*, after analyzing the effects of six plant extracts in *C. elegans*, postulated that a direct antioxidant effect is unlikely to be the main factor responsible for the modulation of nematode lifespan [22].

**Figure 6:**
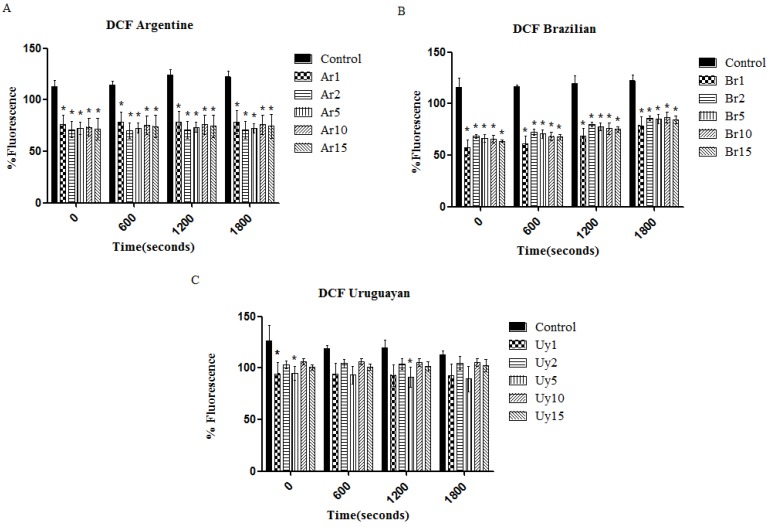
*Ilex paraguariensis* extracts reduce the ROS levels. (**A**) Argentine herb; (**B**) Brazilian herb; (**C**) Uruguayan herb. Data are expressed mean±SEM. * indicates *p* < 0.05 as compared to controls.

Although there are opposing results and opinions, our results showed that associated to this increase in lifespan, there was also a decrease in ROS levels *per se*. All extracts of Argentine and Brazilian yerba mate exerted antioxidant activity by diminishing the quantities of ROS as measured by the DCF-DA method (Figure 6), supporting our hypothesis that this effect is at least partly responsible for the increased lifespan.

### 3.4. The Extracts Increased the Migration of DAF-16 into the Nucleus

Furthermore, we observed that yerba mate extracts caused increased migration of the transcription factor DAF-16 to the nucleus. Taking into account that the beneficial effects of the extracts in relation to stress resistance and longevity were maintained due to the presence of high amounts of polyphenolic compounds, we believe that this could be attributed to an interaction between molecular pathways and scavenger effects of the extracts.

DAF-16 is a transcription factor homologous to the human FOXO, belonging to the IGF-1/insulin-like signaling pathway, which is the central determinant of the endochrine control of stress response, aging, fat metabolism, fertility, and diapause in *C. elegans*. When the worm is submitted to stress or caloric restriction situations, DAF-16 migrates into the nucleus and may modulate the transcription of antioxidant enzymes such as SOD-3, CTL-1 and CTL-2 [10,12,23].

**Figure 7:**
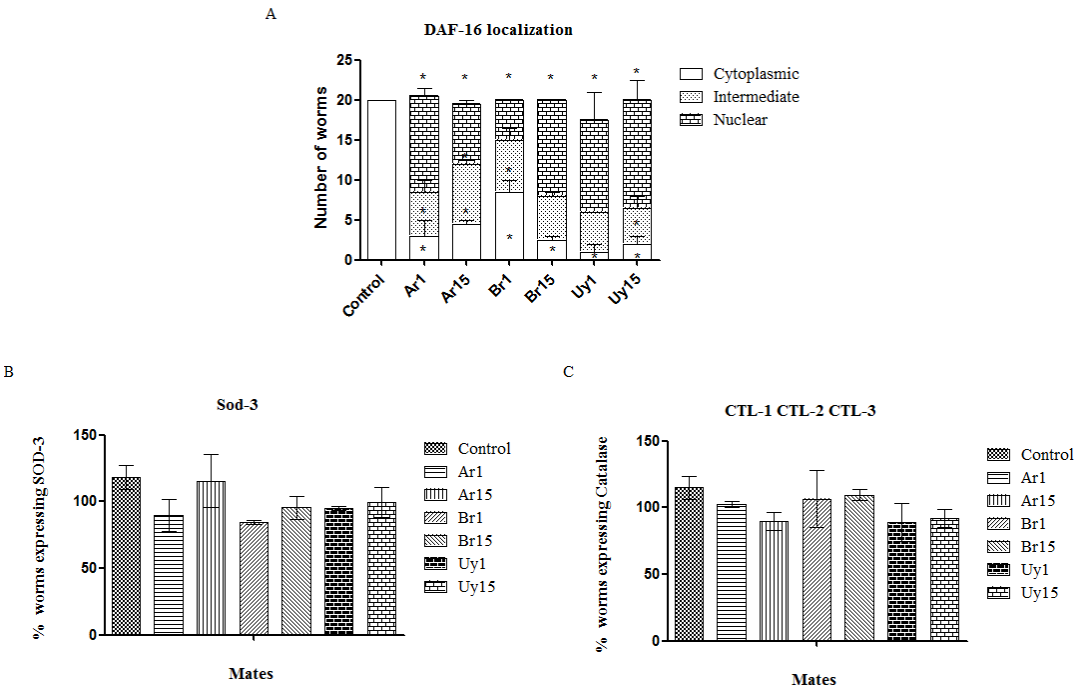
*Ilex paraguariensis* extracts’ effects on the nuclear migration of transcriptional factor DAF-16 (**A**), fluorescence intensity of SOD-3 (**B**) and CTL1; CTL-2;CTL-3 (**C**) in *C. elegans*. Figure 7 A: * indicate *p* < 0.05 compared to the same location of the control group, Data are expressed as mean ± SEM.

We used a strain which has a GFP transgenically fused to the DAF-16 to verify whether the treatment with the *Ilex paraguariensis* extracts would increase the factor translocation to the nucleus of the cell, and in turn, the target genes, responsible for the increased lifespan and resistance to stress in *C. elegans* would be transcribed. Interestingly, we noted that the same extracts that significantly increased the lifespan of *C. elegans* also increased the translocation of DAF-16 to the nucleus (Figure 7A). On the other hand, the exposure to the different extracts did not increase the expression of SOD-3 and catalase antioxidant enzymes, which are antioxidant enzymes that are targets of DAF-16 (Figure 7B,C). DAF-16 also receives input from various other pathways that regulate lifespan, such as the JNK pathway [24], and these pathways may be assisting in these effects.

Buchter *et al.* found that the flavonoid myricetin significantly increased the life of the nematode *C. elegans* [25], and in consonance with our results, concluded that the effect of myricetin in *C. elegans* lifespan is dependent on DAF-16 and not mediated by anti-oxidative property of the flavonoid. Similarly, Guha *et al.* suggested that cranberry supplementation confers increased longevity and stress resistance in *C. elegans* through pathways modulated by DAF-16 and OSR-1 (osmotic stress resistant-1) [26]. Since the insulin/IGF-like signaling (IIS) cascade is a key regulator of lifespan, Grunz *et al*. tested isolated structures of some flavonoids and found an increased DAF-16 translocation and SOD-3 promoter activity were observed with all flavonoids but was independent of their ROS scavenging capability and their effects on lifespan [27]. In our study, we did not observe the induction of SOD-3 transcription and worms lacking daf-16 treated with the extracts depicted the same protection effect against paraquat-induced toxicity. Therefore, we suggest that the components of yerba mate are responsible for the protective effects reported here, possibly by scavenging the ROS produced or by modulating another pathway that was not investigated in this study.

## 4. Conclusions

In summary, our results showed that extracts *Ilex paraguariensis* of different origins extracted in the same as way people habitually consume yerba mate, were able to increase the lifespan of the nematode *C. elegans*, an effect that was associated to the reduction of ROS levels and the possible antioxidant activity of infusions *per se*.

Furthermore, the extracts protected against the toxic effects of paraquat, which is a superoxide generator and a very toxic pesticide. Notably, although some extracts have high phenolic composition, they did not show an increase in *C elegans* lifespan.

We suggest that the effects mentioned above may result from a synergy between the components of yerba mate, such as its phenolic compounds and others not investigated. As the migration of DAF-16 to the nucleus did not increase SOD and CAT levels, this pathway is not responsible for the modulation of the protective effects that the extracts exert. We believe that the herb has potent antioxidant activity, not requiring activation of SOD and CAT enzymes. Thus, in the future, we will examine other molecular pathways and/or genes and verify if they are related to the effects reported in this manuscript.
